# Case Report: Pitfalls in CF screening – targeted variant analysis can cause misleading results and therapy recommendations

**DOI:** 10.3389/fgene.2025.1693573

**Published:** 2025-12-01

**Authors:** Maike Karnstedt, Simone Ahting, Sophie Behrendt, Maike vom Hove, Julia Hentschel

**Affiliations:** 1 Institute of Human Genetics, University of Leipzig Medical Center, Leipzig, Germany; 2 Department of Pediatrics, University of Leipzig Medical Center, Leipzig, Germany

**Keywords:** complex allele, newborn screening, next-generation sequencing, CFTR modulators, cystic fibrosis transmembrane regulator (CFTR)

## Abstract

**Background:**

Cystic Fibrosis (CF) is primarily diagnosed in Germany through newborn screening (NS) using immunoreactive trypsinogen (IRT)/Pancreatitis-Associated Protein (PAP) measurements and genetic testing for common CFTR gene variants. While this method is effective in identifying the most frequent mutations, it may overlook complex alleles, which can impact phenotype and treatment efficacy.

**Case Presentation:**

We report the case of a five-year-old girl diagnosed with CF through NS, initially identified as homozygous for the F508del variant. Despite early Orkambi therapy, her response was suboptimal, with high sweat chloride levels and recurrent respiratory infections. Re-sequencing with a next-generation sequencing (NGS) panel revealed an additional undetected heterozygous pathogenic variant. Upon switching to elexacaftor/tezacaftor/ivacaftor (ETI), sweat chloride levels significantly improved.

**Conclusion:**

Standard genetic screening methods may fail to detect complex alleles, leading to misinterpretation of genotype and suboptimal treatment choices. This case highlights the necessity of comprehensive genetic analysis in patients with unexpected therapy responses. When CFTR modulator therapy does not yield the expected improvements, re-sequencing should be considered to optimize precision medicine approaches for CF.

## Introduction

In Germany, Cystic Fibrosis (CF) is diagnosed by Newborn Screening (NS) comprising immunoreactive trypsinogen (IRT)/Pancreatitis-Associated Protein (PAP) measurements, followed by targeted genetic testing for the most common *CFTR* variants (Heinemann et al., 2016). Nowadays, the majority of patients are diagnosed via NS within the first month of their lives (Nährlich et al., 2023).

CF has a strong genotype-phenotype correlation, with variants sorted into seven different classes depending on their pathomechanism (Boeck and Amaral, 2016). Therapy of CF was revolutionised with the implementation of CFTR modulators by directly addressing these molecular defects, showing particular efficacy for class II (misfolding) and III (chloride channel gating) variants (Ensinck and Carlon, 2022). Precise genotyping is therefore essential for selecting the most effective modulator therapy. When identifying the most common variant F508del in a homozygous state, the combination of lumacaftor and ivacaftor (trade name: Orkambi) is a possible option for treatment starting from 1 year of age. Another therapeutic option consisting of the three compounds elexacaftor (E), tezacaftor (T) and ivacaftor (I) (ETI, trade name: Kaftrio/Trikafta) is approved for people with CF (pwCF) 2 years and older carrying at least one F508del variant.

A complex allele arises when multiple variants are located *in cis* on the same *CFTR* allele. For *CFTR*, the presence of a second variant additionally to the *in-cis*-variant can have impact on the clinical phenotype (El-Seedy and Ladeveze, 2024). However, little is known about the general epidemiology and functional relevance of complex alleles. A systematic evaluation from Russia identified 8,2% of pwCF with the complex allele [L467F; F508del] in F508del homozygous patients (Kondratyeva et al., 2022), characterised by an additional missense substitution [p. (Leu467Phe)] *in-cis* to F508del. This complex allele in homozygous state showed reduced response to ETI in intestinal organoids (Efremova et al., 2024).

Using standard genetic screening methods, complex alleles such as [L467F; F508del] would be missed completely. Therefore, testing for only the most common variants can lead to false results followed by ineffective therapeutic recommendations.

Here we present a case of now five-year-old girl, initially diagnosed with CF by NS and DNA testing, identifying homozygosity for the most common F508del variant in *CFTR,* with no other variant detected. Using our previously described next-generation (NGS) sequencing panel for identification of variants in the entire *CFTR* locus (Ahting et al., 2024), we subsequently identified another heterozygous pathogenic variant that was previously missed (Paolis et al., 2023; Chevalier and Hinzpeter, 2020).

## Clinical presentation

**FIGURE 1:**
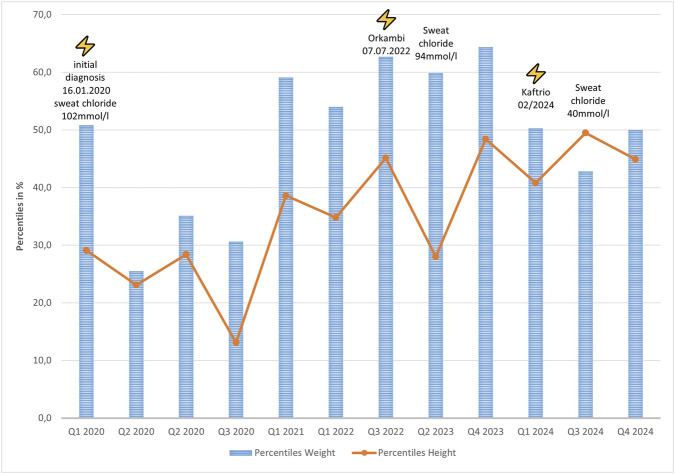
Weight (blue) and height (orange) percentile development of the patient with starting points of modulator therapy and measured sweat chloride concentrations.

The girl was born in 2020 after uneventful pregnancy (born 38+2 weeks, weight: 3,34 ± 0,07 kg, height: 49 ± 0.93 cm, head circumference: 33,5 ± 0,7 cm). CF diagnosis was drawn at postnatal day 12, after positive IRT-PAP-DNA screening (F508del homozygous, Figure 1A) and confirmation by sweat chloride testing (102 mmol/L). Pancreatic elastase was below 50 μg/g faeces, so pancreatic insufficiency was diagnosed and treatment with enzyme substitution was started immediately. Treatment with Orkambi was initiated in July 2022. While she developed normally (Figure 1), sweat chloride levels were only reduced to 94 mmol/L, still in the highly pathological range. The patient suffered of recurrent airway infections. In 2024 at the age of four, modulator therapy was switched to ETI due to its approval. Weight and height development was still normal, while sweat chloride levels reduced to 40 mmol/L. Pancreatic insufficiency persisted in the presence of modulator therapy so far. Her FEV_1_ was measured at 122%, indicating an above-average pulmonary function.

## Genetic testing results

**FIGURE 2:**
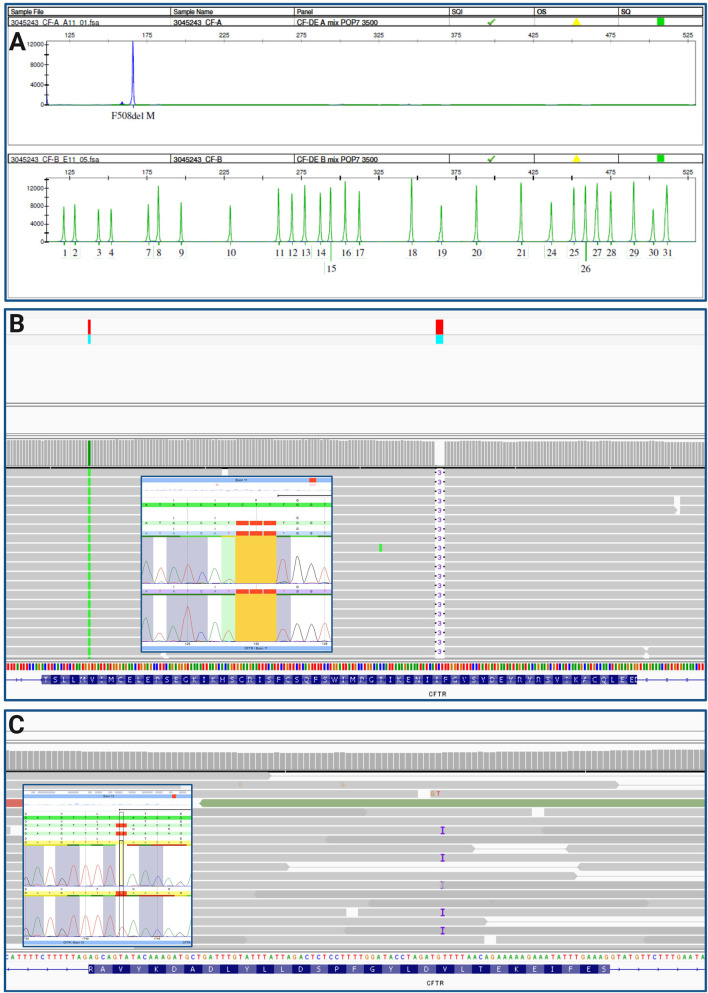
**(A)** CF Elucigene assay, 31 most common variants in *CFTR* were tested, detected F508del homozygous (blue peak, upper panel), green peaks indicate absence of a variant; **(B)** IGV trace of F508del variant homozygous and Sanger sequencing results; **(C)** IGV trace of variant **(C)** 1742dup, p. (Leu581Phefs*8) and according Sanger sequencing results.

For diagnosis of CF, genetic testing was performed at the age of 19 days via CF Strip Assay^®^ (ViennaLab, Vienna, Austria), according to manufacturer’s instructions. We identified the homozygous pathogenic variant NM_000492.4: c.1521_1523delCTT, p. (Phe508del) (legacy name F508del) and confirmed it using the Elucigene CF kit for the detection of the 31 most frequent variants in the European population (Elucigene®, Delta Diagnostics, Manchester, United Kingdom, according to manufacturer’s instructions) (Figure 2A, upper panel). The modulator Orkambi was prescribed to the individual on the assumption of the homozygous state of the F508del variant. Within a research project investigating frequency and relevance of complex alleles, we were looking into pwCF responding well to ETI treatment therapy and not carrying a complex allele (responder positive controls). When resequencing the individual using our NGS custom panel (Ahting et al., 2024), we detected an additional heterozygous variant NM_000492.4:c.1742dup, p. (Leu581Phefs*8) in exon 13 of *CFTR* (Figure 2B). To confirm these findings, Sanger sequencing was conducted (Figure 2C). The complex allele carrying the two variants [p. (Phe508del); p. (Leu581Phefs*8)] will most likely be degraded via nonsense-mediated mRNA decay (NMD), due to the introduction of a premature stop codon, resulting in a class I variant. This type of variants cause the assembly of the CFTR protein to be stopped prematurely, so that no CFTR channel can be formed at all (Bell et al., 2020). Variants were classified according to the latest criteria of the American College of Medical Genetics and Genomics (Richards et al., 2015), as well as ACGS Best Practice Guidelines for Variant Classification 2024 (Durkie et al., 2025) using Varvis software (Limbus, Rostock, Germany).

## Conclusion

Through the pathomechanism of the complex allele, the therapy with Orkambi led to fewer improvements as expected at the beginning of treatment. Screening for the most common variants is cost-effective and provides results within a few days. However, since this approach does not detect all variants, it may miss complex alleles, and ultimately result in ineffective therapy. Whenever patients do not respond to modulators as expected, a re-sequencing should be considered. Furthermore, discontinuation of modulator therapy should be discussed and standard CF therapy should be continued. Newborn genomic screening (NBGS) offers a promising expansion of conventional programs by enabling broader detection of pathogenic variants and accelerating diagnosis. While current NBGS technologies cannot yet fully replace traditional methods, integrating both approaches can substantially improve screening accuracy, efficiency, and equity across populations. Recent advances in cystic fibrosis screening illustrate this progress, as expanded CFTR variant panels and next-generation sequencing now support more inclusive and timely detection, moving toward primary genetic screening (Men et al., 2025; Tariq et al., 2025; Farrell, 2025).

## Data Availability

All relevant data is contained within the article: The original contributions presented in the study are included in the article, further inquiries can be directed to the corresponding author.

## References

[B1] Ahting S. Nährlich L. Held I. Henn C. Krill A. Landwehr K. (2024). Every CFTR variant counts - target-capture based next-generation-sequencing for molecular diagnosis in the German *cf* registry. J. Cystic Fibrosis Official Journal Eur. Cyst. Fibros. Soc. 23 (4), 774–781. 10.1016/j.jcf.2023.10.009 37867076

[B2] Bell S. C. Mall M. A. Gutierrez H. Macek M. Madge S. Davies J. C. (2020). The future of cystic fibrosis care: a global perspective. Lancet. Respir. Medicine 8 (1), 65–124. 10.1016/S2213-2600(19)30337-6 31570318 PMC8862661

[B3] Boeck K. de Amaral M. D. (2016). Progress in therapies for cystic fibrosis. Lancet. Respir. Medicine 4 (8), 662–674. 10.1016/S2213-2600(16)00023-0 27053340

[B4] Chevalier B. Hinzpeter A. (2020). The influence of CFTR complex alleles on precision therapy of cystic fibrosis. J. Cystic Fibrosis Official Journal Eur. Cyst. Fibros. Soc. 19 (Suppl. 1), S15–S18. 10.1016/j.jcf.2019.12.008 31883651

[B5] Durkie M. Cassidy E.-J. Berry I. Owens M. Turnbull C. Scott R. H. (2025). “ACGS best practice guidelines for variant classification in rare disease,” in Association for clinical genomic science. Available online at: https://www.acgs.uk.com/quality/best-practice-guidelines/17 October, 2025).

[B6] Efremova A. Melyanovskaya Y. Krasnova M. Voronkova A. Mokrousova D. Zhekaite E. (2024). Estimation of chloride channel residual function and assessment of targeted drugs efficiency in the presence of a complex allele L467F;F508del in the CFTR gene. Int. Journal Molecular Sciences 25 (19), 10424. 10.3390/ijms251910424 39408749 PMC11476812

[B7] El-Seedy A. Ladeveze V. (2024). CFTR complex alleles and phenotypic variability in cystic fibrosis disease. Cell. Molecular Biology (Noisy-le-Grand, France) 70 (8), 244–260. 10.14715/cmb/2024.70.8.33 39262237

[B8] Ensinck M. M. Carlon M. S. (2022). One size does not fit all: the past, present and future of cystic fibrosis causal therapies. Cells 11 (12), 1868. 10.3390/cells11121868 35740997 PMC9220995

[B9] Farrell P. M. (2025). Reflections on 50 years of cystic fibrosis newborn screening experience with critical perspectives, assessment of current status, and predictions for future improvements. Int. Journal Neonatal Screening 11 (4), 88. 10.3390/ijns11040088 41133700 PMC12551092

[B10] Heinemann M. L. Hentschel J. Becker S. Prenzel F. Henn C. Kiess W. (2016). Einführung des deutschlandweiten Neugeborenenscreenings für Mukoviszidose. LaboratoriumsMedizin 40 (6), 373–384. 10.1515/labmed-2016-0062

[B11] Kondratyeva E. Efremova A. Melyanovskaya Y. Voronkova A. Polyakov A. Bulatenko N. (2022). Evaluation of the complex p.Leu467Phe;Phe508del CFTR allele in the intestinal organoids model: implications for therapy. Int. Journal Molecular Sciences 23 (18), 10377. 10.3390/ijms231810377 36142302 PMC9499621

[B12] Men S. Wang Z. Liu S. Tang X. Liu S. Zhao Y. (2025). Integrating newborn genetic screening with traditional screening to improve newborn screening. J. Matern. Fetal Neonatal Med. 38 (1), 2583588. 10.1080/14767058.2025.2583588 41198170

[B13] Nährlich L. Burkhart M. Wosniok J. (2023). German cystic fibrosis registry annual report 2023.

[B14] Paolis E. de Tilocca B. Lombardi C. Bonis M. de Concolino P. Onori M. E. (2023). Next-generation sequencing for screening analysis of cystic fibrosis: spectrum and novel variants in a south-central Italian cohort. Genes 14 (8), 1608. 10.3390/genes14081608 37628659 PMC10454170

[B15] Richards S. Aziz N. Bale S. Bick D. Das S. Gastier-Foster J. (2015). Standards and guidelines for the interpretation of sequence variants: a joint consensus recommendation of the American college of medical genetics and genomics and the association for molecular pathology. Genet. Medicine Official Journal Am. Coll. Med. Genet. 17 (5), 405–424. 10.1038/gim.2015.30 25741868 PMC4544753

[B16] Tariq H. Salpietro V. Houlden H. Stevanin G. (2025). Globalizing newborn screening: bridging gaps in genetic diagnosis and treatment. Front. Pediatrics 13, 1672993. 10.3389/fped.2025.1672993 41181182 PMC12571786

